# Intranasal fentanyl for respiratory distress in children and adolescents with life-limiting conditions

**DOI:** 10.1186/s12904-018-0361-x

**Published:** 2018-09-10

**Authors:** Lucas Pieper, Julia Wager, Boris Zernikow

**Affiliations:** 1 Department of Children’s Pain Therapy and Paediatric Palliative Care, Witten/Herdecke University, Faculty of Health – School of Medicine, Witten, Germany; 2 Paediatric Palliative Care Centre, Children’s and Adolescents’ Hospital Datteln, Dr.-Friedrich-Steiner-Str.5, 45711 Datteln, Germany

**Keywords:** Dyspnea, Fentanyl, Intranasal application, Palliative, Pediatric

## Abstract

**Background:**

Respiratory distress is one of the most common and frightening symptoms of children with life-limiting conditions. Because treatment of the underlying cause is frequently impossible or insufficient, in many children, symptomatic treatment is warranted. The purpose of this study was to describe the circumstances of the use of intranasal fentanyl in an acute attack of respiratory distress (AARD) in children receiving palliative care, as well as to describe outcomes and adverse events after its use.

**Methods:**

Children and adolescents treated in a pediatric palliative unit or attended by a specialized home care team between 2010 and 2016 were included in this study. A retrospective chart review was conducted of those who were treated with intranasal fentanyl for an AARD.

**Results:**

During the study period 16 children (0.5–18.6 years) with various life-limiting conditions were treated with intranasal fentanyl for AARD. In total, 70 AARDs were analyzed. In 74% of all AARDs, a single dose of intranasal fentanyl was used. Frequent causes for an AARD were excessive secretions and acute respiratory infection. The median starting dose of intranasal fentanyl was 1.5 μg/kg body weight. Labored breathing (96%), tachypnea (79%) and related suffering (97%) improved after treatment. An adverse event occurred in one child.

**Conclusions:**

Intranasal fentanyl may be a safe and effective medication for the treatment of acute attacks of respiratory distress in children with life-limiting conditions. However, prospective studies with larger sample sizes and a control group are needed to validate these findings.

## Background

Respiratory distress (RD) is a concerning symptom with high prevalence in children with life-limiting conditions (LLCs) [1–15]. RD is frequently reported in cancer, advanced heart or pulmonary disease, neurological disorders and many other rare LLCs [1–8, 10–12, 14]. The management of RD is therefore an important part of pediatric palliative care.

In children and adolescents of all ages, RD can occur with a wide range of physical signs and unpleasant perceptions [16]. The latter are often described as dyspnea, “a subjective experience of breathing discomfort that consists of qualitatively distinct sensations that vary in intensity” [17]. The multidimensional concept of dyspnea describes the complex pathophysiology of this symptom with interacting biological, psychological, social and environmental factors. Numerous chemo- and mechanoreceptors as well as neural pathways are involved in this condition [17–19]. RD can also be caused by increased respiratory drive, mechanical impedance, respiratory muscle failure, wasted ventilation and psychological stress [19] causing sensations such as air hunger, a sense of increased work of breathing, labored breathing and chest tightness [20, 21].

The most effective treatment of RD is the identification and therapy of underlying causes. However, this approach is frequently limited when no specific cause is apparent or no effective treatment at hand [22]. In this situation, symptomatic treatment is warranted. Non-pharmacological approaches include physiotherapy, management of secretions, fans to blow in the face, psychological and behavioral therapy and (noninvasive) positive pressure ventilation [1, 22, 23]. Pharmacological treatment mainly consists of opioids and anxiolytics [1, 22, 24]. In the pharmacological treatment of RD in adults, opioids are used frequently with good evidence for the oral and parenteral route [24, 25].

One treatment approach is intranasal fentanyl (INF); it is used to treat dyspnea in adults with chronic obstructive pulmonary disease, chronic heart failure and lung cancer [26, 27]. Fentanyl is a lipophilic opioid and can therefore easily traverse the highly vascularized and permeable nasal mucosa [28]. Intranasal drug delivery is described as an effective and non-invasive approach for systemic administration [28]. In pain treatment, the onset of action occurs within 5 min after the application of INF [29]. This is very short, compared to the long onset of action of 15–30 min when short acting morphine is given orally [30]. INF also avoids the hepatic first pass effect [28]. In one study, INF treatment led to significantly higher fentanyl plasma levels and had a higher bioavailability than the oral-transmucosal administration of fentanyl [31].

In pediatric pain management INF is a rather new treatment approach; it has been used with good evidence postoperatively and in acute pain due to burns or fractures [32–34]. Although evidence for the effectiveness of INF administration for RD is scant [35, 36], clinical experience indicates a safe reduction of symptom intensity and related suffering [26, 37, 38]. A case series with 11 newborns and infants describes the good effectiveness and tolerability of INF for the palliation of RD, without the occurrence of apnea or chest wall rigidity [37]. Nonetheless, the use of INF for the palliation of RD in children requires further exploration, specifically the use in various age groups and for various underlying diseases. Solid knowledge regarding the circumstances of administration, dosage, outcomes, safety and adverse effects needs to be generated.

The primary purpose of this study is to report the application of INF for RD treatment in a sample of palliative attended children and adolescents with various LLCs based on data from a retrospective chart analysis. First, the symptoms and conditions leading to the administration of INF are described. Second, the extent and frequency in which INF has been used during acute attacks of RD are presented. Third, the therapeutic response and potential adverse events are analyzed.

## Methods

### Study design and sample

We performed a retrospective chart review of pediatric in- and outpatients, who were either treated in a pediatric palliative ward or by a specialized pediatric palliative home care team. From June 2010 to July 2016, 402 patients with LLC were treated inpatient, and 198 patients were treated by the home care team. All children received palliative care in various phases of their illness trajectory. A prior study showed that approximately 30% of inpatients suffer from RD [14].

Patients were considered eligible for the study if they were treated with INF for RD. Those treated with INF were detected via the controlled drugs register (*n* = 28). In 57% of the patients (*n* = 16), INF was used for the palliation of RD. In the remaining 43% of the patients (*n* = 12), the drug was administered for breakthrough pain control.

### Data collection

A retrospective chart review was conducted for the 16 eligible patients. Both, electronic and handwritten medical charts, pharmacy data and doctor’s letters were screened for this research. The documentation did not change over time. At all time points, electronic and handwritten charts were used in parallel for the documentation of different aspects. All documents were screened for indicators of an acute attack of respiratory distress (AARD). For this purpose, descriptions and acronyms of RD and associated conditions such as suffering and restlessness were searched in the documents (Table 1). A mean of 2 and a maximum of 5 different verbal descriptions of RD were documented during an AARD.

**Table 1:** Indicators for dyspnea: searched terms for symptoms and general conditions

symptoms/condition	descriptions in the documents
respiratory distress	“labored breathing” incl. “chest wall retractions”, “use of accessory muscles”, “nasal flaring”, “heavy breathing”, “difficult breathing”
	“tachypnea”
	“dyspnea”^b^
	“wheezing”
	“cyanosis”
suffering	“suffering”, “sorrow”, “crying”
restlessness	“is restless”
exhaustion	“is weak”, “is exhausted”

For each AARD, symptoms and conditions leading to the administration of INF were extracted. Additionally, the continuous medication and adjunctive therapies for RD were documented.

To measure the therapeutic response of the INF application, any reported improvement of RD, RD-associated symptoms, suffering and restlessness were ranked on a 4-point scale (0 = no, 1 = slight, 2 = moderate, and 3 = complete resolution). For that purpose, the researchers collected typifying terms of the different levels of improvement (no/ slight/ moderate/ complete resolution) in the medical charts. The assignment of these terms to the 4-point scale was carried out in an interdisciplinary palliative care team consisting of physicians, psychologists and nurses. Following, a researcher (LP) assessed any reported improvement of RD in an AARD guided by the collections of typifying terms. The ranking could only be applied if the same symptom was reported prior to the INF application and after treatment. A symptom was considered resolved if it was reported prior to the INF application and not reported afterward. Vital signs such as heart rate, oxygen saturation, respiratory rate and blood pressure before and after the administration were collected. However, vital signs were not documented in all AARDs; they are regarded with rather low importance in pediatric palliative care (PPC). Due to the small number of usable findings, they are not reported in this manuscript.

We recorded the circumstances of every single INF application, number of doses of INF applied per AARD, the cumulative dosage and the length of intervals between 2 INF doses. The end of an AARD was defined as the absence of signs of RD occurring for at least 1.5 h. The adverse effects occurring in association with INF application were documented.

### Statistical analysis

Analyses were conducted with the SPSS 24 statistical software program. The results of the descriptive statistics are reported as the total number (n) and percentages (%), as well as the median and the range of values or mean and standard deviation (SD). A comparison regarding the initial dose of INF between opioid naïve children and non-naïve children was calculated with a Mann-Whitney U-Test. A significance level of *p* < .05 is considered significant.

Results are reported separately for 1) the first dose of INF (*n* = 70), 2) the second dose of INF (*n* = 18) and 3) the last dose of INF (*n* = 70). The section “last dose of INF” deals with the final dose of all reviewed AARD (n = 70, 100%) and also includes all AARD treated with only one dose of INF. For the first dose, circumstances prior to administration, details of treatment and treatment success are reported. For the second dose, only details on treatment are presented, while for the last dose, only the treatment success is reported.

## Results

### Patient characteristics

The characteristics of all 16 patients included in this study are summarized in Table 2. Nine patients were male and seven were female. The age of patients ranged from 0.5 to 18.6 years. The median patient age was 5.9 years. All patients had different diagnoses, except for three children who suffered from spinal muscular atrophy type I and two patients from hypoxic-ischemic encephalopathy. Thirteen children (81%) were unable to self-report RD or related suffering due to young age or cognitive impairment.

**Table 2:** Sample characteristics

*N* = 16	*n*	%
sex		
male	9	56
female	7	44
setting		
outpatient	5	31
inpatient	10	63
inpatient+ outpatient	1	6
underlying condition		
acute lymphoblastic leukemia	1	
alveolar rhabdomyosarcoma	1	
chronic granulomatous disease, Graft-versus-host disease	1	
Cockayne syndrome	1	
global developmental delay of unknown origin	1	
hypoxic-ischemic encephalopathy	2	
Menkes disease, prematurity	1	
neurodegenerative disease of unknown origin	1	
neuronal ceroid lipofuscinosis type 3	1	
Prune belly syndrome, periventricular leukomalacia	1	
spinal muscular atrophy type 1	3	
Tetralogy of Fallot, hypoxic ischaemic encephalopathy	1	
Trisomy 13	1	

Note: percentages are rounded and may therefore add up to more than 100%

### General description of intranasal fentanyl application

In the 16 patients, 70 AARDs (100%) were treated with 122 INF applications. In total, 38 (54%) of the AARDs occurred in opioid naïve children, and 32 (46%) occurred in children already receiving opioids via various administration routes on a daily basis. The median daily oral morphine equivalent dose of the continuous opioid application was 0.72 mg/kg body weight (bw) (range 0.1–12.24 mg/kg bw/day).

**Fig. 1:**
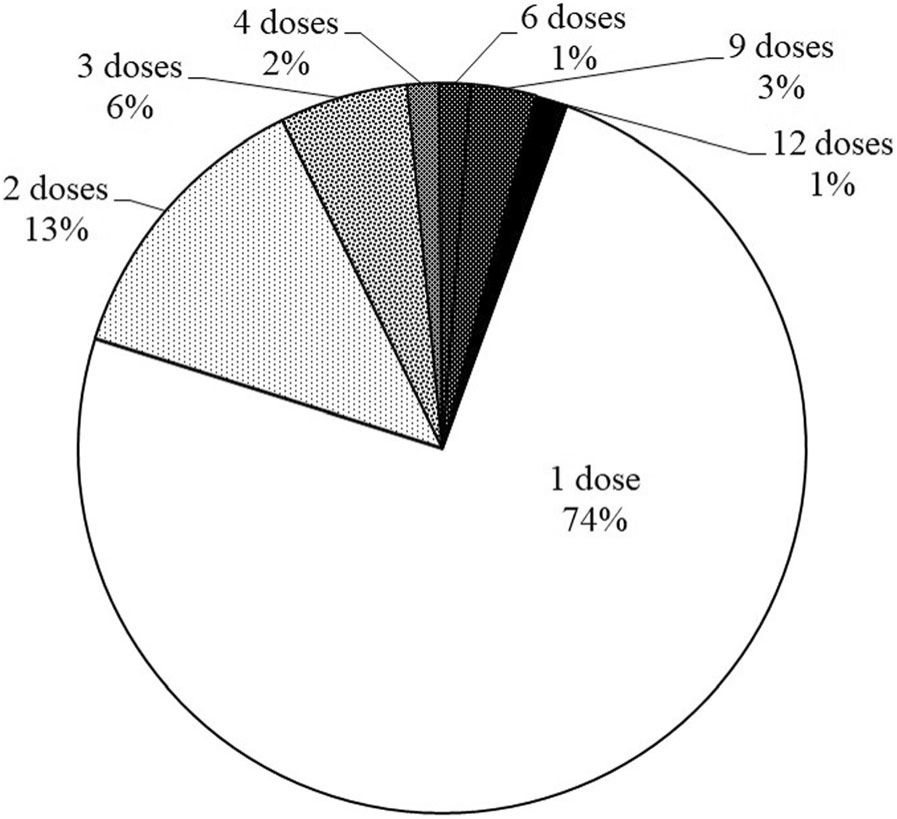
Number of INF doses in a single acute attack of respiratory distress. Note: percentages are rounded and add up to less than 100%

The mean number of administered doses in a single AARD was 1.74 (SD = 1.98). One child with infectious hypoxic encephalopathy received 12 doses in a single AARD during his terminal phase, but the majority (*n* = 52, 74%) of attacks were treated with a single dose of INF (Fig. 1). The used drugs were either 1) nebulized intravenous fentanyl (50 μg/ml) for lower doses or 2) Instanyl (500-2000 μg/ml) for higher doses.

## First dose of INF

### Circumstances prior to administration

Various potential reasons for RD were recorded (Table 3). Excessive secretion was documented in 70% of all AARDs. Furthermore, in 67% of AARDs, an acute respiratory infection was documented when the first dose of INF was administered. Other, less frequently described pathophysiologies were anxiety, pain, pleural effusion, atelectasis and hepatosplenomegaly.

**Table 3:** Documented circumstances prior to the first administration of intranasal fentanyl (*n* = 70)

Circumstances prior to administration	number of AARDs	
	*n*	%
underlying pathophysiology		
excessive secretion	49	70
respiratory infection	47	67
muscular hypotonia	19	27
limited ability to cough	14	20
lower airway obstruction	14	20
upper airway obstruction	4	6
others^a^	14	20
general condition		
suffering	34	49
restlessness	29	41
exhaustion	13	19
sleepiness	12	17
description of respiratory distress		
labored breathing	47	67
tachypnea	41	59
dyspnea	27	39
wheezing	7	10
cyanosis	6	9

Note: percentages are rounded; multiple responses possible

^a^others includes: atelectasis, pleural effusion, anxiety

The initial usage of INF was connected in timely fashion to the occurrence of various descriptions of RD and the impairment of the general condition (Table 3). In the majority of AARDs, several signs of RD appeared simultaneously. The most prevalent descriptions prior to the first administration of INF was labored breathing (67%), followed by tachypnea (59%). “Dyspnea” as a descriptor was documented in 39% of all AARDs. General suffering was reported in nearly half of all AARDs. In 41% of the children, restlessness was reported.

### Treatment of an AARD

The median starting dose of INF was 40 μg (range 10–100 μg), which equals 1.5 μg/kg bw (range 0.4 μg–5.1 μg/kg bw). Opioid-naïve children were initially treated with a median dose of 0.97 μg/kg bw (range 0.42–1.5 μg/kg bw), while children already receiving opioid treatment started with a significantly higher dose of 1.7 μg/kg bw (range 0.54–5.1 μg/kg bw) (*p* < .001; U-Test).

In addition to INF application, various approaches of adjunctive therapy were used to improve the patient’s respiratory situation (Table 4). Continuous medication for RD palliation was applied in 67 AARDs (96%). Most common were inhaled salbutamol (74%) and ipratropium bromide (39%). Other continuous medications for RD were inhalations with NaCl 0.9%, salmeterol and budesonide or treatment with opioids, glucocorticoids, furosemide and xylometazoline. In 81% of all AARDs, oxygen was applied initially with a range from 2 to 15 l and a median of 4.5 l. Additional on-demand medication was used in 4 AARDs (6%).

**Table 4:** Adjunctive therapy administered when the first dose of intranasal Fentanyl was given (*n* = 70)

adjunctive therapies	number of AARDs	
	*n*	%
continuous medication		
Budesonide, inhalation	12	17
Furosemide, orally/intravenously	13	19
Glucocorticoids, orally/intravenously	15	21
Ipratropium bromide, inhalation	27	39
Levamethadone, orally	4	6
Morphine, patient controlled analgesia	7	10
Morphine, continual intravenous infusion	2	3
Morphine, slow release, orally	19	27
NaCl 0.9%, inhalation	25	36
Salbutamol, inhalation	52	74
Salmeterol/Fluticasone, inhalation	20	29
Xylometazoline, intranasally	8	11
Other medications	3	4
on demand medication		
Ipratropium bromide, inhalation	3	4
Salbutamol, inhalation	1	1
Oxygen	57	81
non-pharmacological treatments		
mechanical suction of secretion	38	54
physiotherapy	11	16
others	4	6

Note: Percentages add up to more than 100% because multiple responses are possible

### Treatment success

**Fig. 2:**
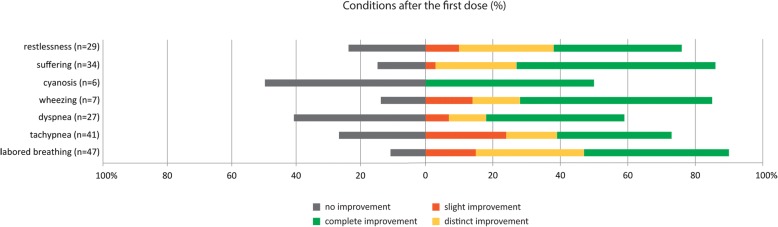
Conditions after the first intranasal fentanyl dose. Note: rounded percentages

Figure 2 displays the effects of INF application for the various symptoms and conditions. The administration of the first INF dose in an AARD had the greatest effect on “labored breathing” (improved in 89%) and “tachypnea” (improved in 73%). When the term “dyspnea” was documented, it improved in more than half of all AARDs after the first INF application (59%). With regard to the general condition of the patient, “suffering” and “restlessness” were reduced in 85% and 76% of all AARDs after the first INF dose, respectively.

### Adverse events

In just one case, an adverse event was documented after the first INF dose. The affected child had Trisomy 13, with body weight of 55 kg. The child was treated with a starting dose of 50 μg (0.9 μg/kg bw) of INF during “labored breathing”, “suffering” and “restlessness”. After the INF administration, Cheyne-Stokes respiration occurred, and a decrease in the oxygen saturation was documented. The only continuous drugs admitted at the time were furosemide and ipratropium bromide inhalation. After the administration of oxygen (10 l/min) the oxygen saturation improved. The Cheyne-Stokes respiration disappeared shortly afterward. In this child, four administrations of similar INF doses in the days prior and six applications after this event occurred without any recorded side effects. The described adverse effect was not noted with other doses of INF. Further adverse effects like chest wall rigidity did not occur.

## Second dose of INF

In 18 (26%) AARDs, a second dose of INF was administered. The time interval between the first and second dose ranged from 15 to 80 min, with a median interval of 30 min. For all patients, the amount of the second INF dose was the same as that of the first dose. The median dose was 1.3 μg/kg bw (range 0.67–4.08 μg/kg bw).

## Last dose of INF

**Fig. 3:**
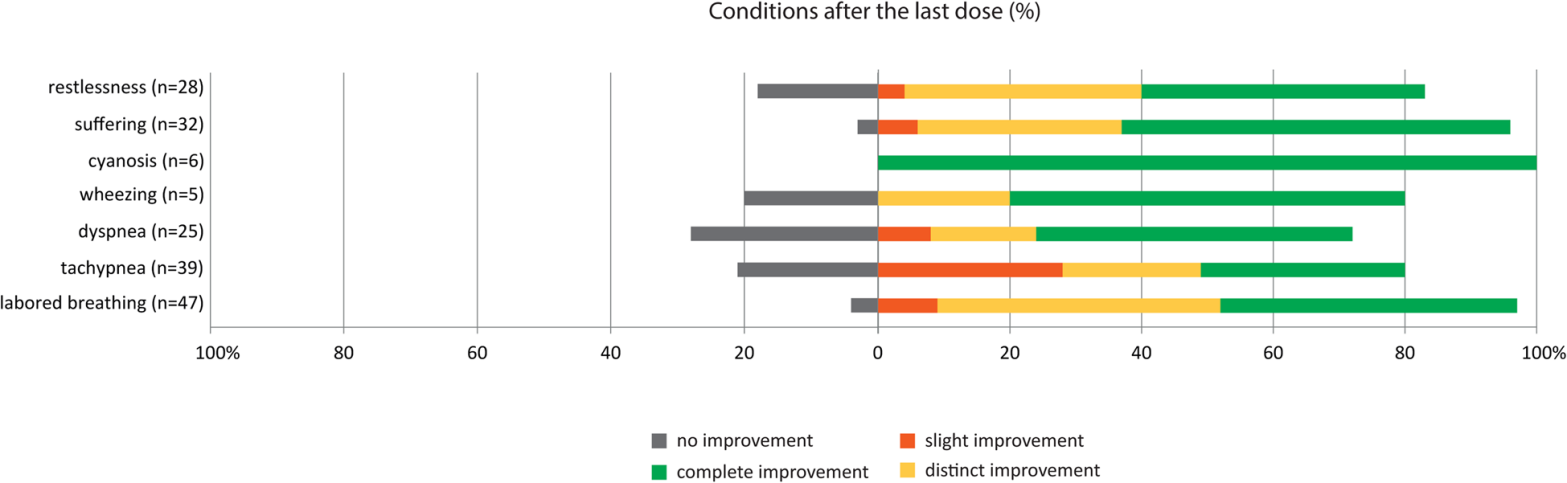
Conditions after the last dose of intranasal fentanyl dose. Note: rounded percentages

The cumulative INF dose in one AARD (median 50 μg) ranged from 10 μg in two toddlers to 500 μg in a 19-year-old with neuronal ceroid lipofuscinosis type 3 in the terminal phase. The highest rate of improvement was achieved when “labored breathing” (96%) and “tachypnea” (79%) occurred before the last administration of INF (Fig. 3). When the term “dyspnea” was documented just prior to the last INF application, it improved in 72% of the AARDs after the last dose of INF and was completely resolved in 48% of the AARDs. “Cyanosis” was completely resolved in all children after the last dose of INF. An improvement of “suffering” was achieved in 97% of all attacks. There was only one documented AARD in which the child’s suffering did not improve at all, although previous attacks that day were treated successfully.

### Adverse events

No additional adverse events were documented after the last dose of INF.

## Discussion

Beside the previous report of INF in eleven infants and newborns [37], this is the first study that describes the usage of INF for the treatment of RD in children with LLC receiving palliative care. The results show that in many children of various ages and with varying diagnosis, symptoms improve after the first INF application; only a small number of patients require a second or more doses. Furthermore, this study indicates that adverse events after INF application are rare. Taken together, this study offers preliminary evidence that INF is a safe and effective medication for the treatment of RD in children with LLC.

In PPC settings, the assessment of RD is often hindered by patients being unable to self-report. The majority of patients have severe psycho-motor impairments and are not able to report dyspnea [14, 16]. Our results underline the complexity of RD. Various descriptions of RD and related suffering are used to paraphrase the patient’s respiratory condition in the chart. This fact impedes comparability. For future research and for clinical communication, it may be of great help to generate a reliable and valid assessment tool for the measurement of RD in children unable to self-report. Furthermore, the small number of self-assessment tools for communication-competent children needs to be extended and adapted to younger ages and validated for a wide range of underlying diseases [16].

During the retrospective chart review, we also noticed that vital signs are not recorded routinely during AARD. Only in approximately 50% of all AARDs, vital signs were documented prior to the administration of INF. The documentation after INF treatment was even more incomplete. This finding suggests that the clinical assessment of a patient may be more relevant in palliative care than the evaluation of vital signs. However, for research purposes, the documentation of AARD in standard care needs to be improved. An assessment tool for RD could therefore be supplemented by documentation of vital signs optionally, if the situation allows the measurement. The patient’s conditions and quality of life should not be affected by the measurement.

This study supports the notion that underlying conditions leading to RD in children with LLC differ from those in adults, who often suffer from chronic obstructive pulmonary disease, heart failure or pulmonary cancer [39–42]. The children in our study suffer from a wide variety of underlying conditions. Diverse biological and psychological pathophysiologies can be found. Excessive and thick secretions and airway infections are the most frequently reported causes of the AARDs, but pain and anxiety may also contribute to the children’s RD. These findings support the multidimensional concept of dyspnea [17, 18, 43].

Similar to previous research in adults [26, 27] and infants [37], this study highlights the suitability of INF treatment for RD. One dose of INF seems frequently adequate to clinically improve RD. In an AARD, the intranasal route has advantages over the oral or oral-transmucosal administration of fentanyl. The rather quick effect of INF is important for the treatment. An AARD is an emergency situation that requires prompt relief. Because the effect of INF becomes apparent very quickly, it also becomes obvious very quickly if an additional dose is required. In our sample, a median time of 30 min passed until the next dose. The minimum interval between first and second dose was 15 min.

The initial median INF dose of 1.5 μg/kg bw for the treatment of an AARD in children reported in this study corresponds to doses used in pediatric pain management. In pediatric pain management, effective analgesia is reported at a dose of 1–2 μg/kg bw of INF [29, 44–51]. In adults, the use of INF for treatment of dyspnea is reported in a few case reports only. In these case reports, the initial INF dose ranged from 50 to 100 μg in opioid-naïve patients. In patients already on continuous opioids, a four-hour equivalent INF dose leads to relief of RD [26, 27]. The only study available describing the palliation of RD in infants and newborns reports initial doses of 0.2–3.8 μg/kg bw of INF, of which more than one half of the children were treated with 1–2 μg/kg bw of INF [37]. The results of our study show that the doses applied are safe and appeared to be rather effective in RD.

The low number of adverse effects is remarkable and supports the assumption of former studies of INF being a good treatment option for RD [26, 27, 37]. The good safety profile, the ease of use, and the effectiveness of INF [26, 27, 32, 37] enable the application in the in- and outpatient setting. However, this study showed that opioids should be used carefully, as adverse effects can occur even in children used to opioids.

The retrospective design of the study and the lack of standardized documentation and a validated assessment tool for the children’s RD may have negatively affected the data collection. Furthermore, the small sample size of this study limits generalizability.

## Limitations

The validity of the data on the efficacy of INF in an AARD may be affected as the ranking was conducted by one researcher. To prevent any bias in the assessment the 4-point scale was designed by an interdisciplinary palliative care team. It provided collections of typifying terms to guide the assessment-process and ensure consistency.

## Conclusions

Our findings suggest that INF is one option to treat acute RD in children with diverse underlying life-limiting conditions and different ages. Good reasons for the INF treatment are the short onset of action, the low invasiveness, and the easy and safe application in a range of children. However, knowledge regarding the palliation of RD with INF needs to be extended. Prospective studies and a control-group-design would be desirable, though challenging. A standardized and validated tool to measure RD in children unable to self-report would help to measure treatment outcome and to identify a requirement for the pharmacological treatment of RD.

## Abbreviations

AARD: Attack of respiratory distress
bw: Body weight
INF: Intranasal fentanyl
LLC: Life-limiting conditions
PPC: Pediatric palliative care
RD: Respiratory distress
